# The Homeobox Gene *MEIS1* Is Methylated in *BRAF* ^p.V600E^ Mutated Colon Tumors

**DOI:** 10.1371/journal.pone.0079898

**Published:** 2013-11-07

**Authors:** Ashwin A. Dihal, Arnoud Boot, Eddy H. van Roon, Melanie Schrumpf, Arantza Fariña-Sarasqueta, Marta Fiocco, Eliane C. M. Zeestraten, Peter J. K. Kuppen, Hans Morreau, Tom van Wezel, Judith M. Boer

**Affiliations:** 1 Center for Human and Clinical Genetics, Leiden University Medical Center, Leiden, The Netherlands; 2 Department of Pathology, Leiden University Medical Center, Leiden, The Netherlands; 3 Department of Medical Statistics, Leiden University Medical Center, Leiden, The Netherlands; 4 Department of Surgery, Leiden University Medical Center, Leiden, The Netherlands; 5 Department of Pediatric Oncology, Erasmus MC, Sophia Children’s Hospital, Rotterdam, The Netherlands; 6 Netherlands Bioinformatics Center, Nijmegen, The Netherlands; Ohio State University Medical Center, United States of America

## Abstract

Development of colorectal cancer (CRC) can occur both via gene mutations in tumor suppressor genes and oncogenes, as well as via epigenetic changes, including DNA methylation. Site-specific methylation in CRC regulates expression of tumor-associated genes. Right-sided colon tumors more frequently have *BRAF* ^p.V600E^ mutations and have higher methylation grades when compared to left-sided malignancies. The aim of this study was to identify DNA methylation changes associated with *BRAF* ^p.V600E^ mutation status. We performed methylation profiling of colon tumor DNA, isolated from frozen sections enriched for epithelial cells by macro-dissection, and from paired healthy tissue. Single gene analyses comparing *BRAF* ^p.V600E^ with *BRAF* wild type revealed *MEIS1* as the most significant differentially methylated gene (log_2_ fold change: 0.89, false discovery rate-adjusted *P*-value 2.8*10^-9^). This finding was validated by methylation-specific PCR that was concordant with the microarray data. Additionally, validation in an independent cohort (*n*=228) showed a significant association between *BRAF* ^p.V600E^ and *MEIS1* methylation (OR: 13.0, 95% CI: 5.2 - 33.0, *P*<0.0001). *MEIS1* methylation was associated with decreased *MEIS1* gene expression in both patient samples and CRC cell lines. The same was true for gene expression of a truncated form of *MEIS1*, *MEIS1*_*D27*_, which misses exon 8 and has a proposed tumor suppression function. To trace the origin of *MEIS1* promoter methylation, 14 colorectal tumors were flow-sorted. Four out of eight *BRAF* ^p.V600E^ tumor epithelial fractions (50%) showed *MEIS1* promoter methylation, as well as three out of eight *BRAF* ^p.V600E^ stromal fractions (38%). Only one out of six *BRAF* wild type showed *MEIS1* promoter methylation in both the epithelial tumor and stromal fractions (17%). In conclusion, *BRAF* ^p.V600E^ colon tumors showed significant *MEIS1* promoter methylation, which was associated with decreased *MEIS1* gene expression.

## Introduction

Colorectal cancer (CRC) is a frequently occurring malignancy in the Western world with a 5% life-time risk [1] and a high worldwide annual incidence (*n* = 1,200,000) and mortality (*n* = 608,000) [2]. CRC is caused by inactivating mutations in tumor suppressor genes and/or activating mutations in proto-oncogenes. Such mutated oncogenes include *KRAS* and *BRAF* that are mutually exclusive in colon tumors [3] and are both part of the Mitogen Activated Protein Kinase (MAPK) pathway. In this biological route, signals traffic from growth factor receptors present on the cell membrane towards the nucleus and finally cause cell proliferation [4]. The *BRAF* gene encodes for the serine/threonine-protein kinase B-Raf and the most commonly found *BRAF* mutation in CRC is present in exon 15 (c.1799TA) [5]. This mutation leads to substitution of valine by the negatively charged glutamic acid at position 600 (*BRAF* ^p.V600E^) and increased protein kinase activity [6]. As a result, *BRAF* ^p.V600E^ leads to constitutive signaling of the MAPK pathway, independent from upstream growth signals. The *BRAF* ^p.V600E^ mutation frequency increases gradually from the rectum to the right-sided colon rather than showing a left-right dichotomy [7]. Additionally, the *BRAF* ^p.V600E^ mutation is an adverse prognostic factor among patients with CRC [8–13] and is associated with both microsatellite instability (MSI-H) and genome-wide DNA promoter methylation [3,14]. MSI-H is caused by deficient mismatch repair as a result of *MLH1* mutation or promoter methylation [15].

In contrast to DNA mutations, DNA methylation is a form of epigenetic alteration in which the DNA sequence is retained. Methylation occurs at the 5-position of the pyrimidine cytosine, only when followed by the purine guanine. DNA promoter methylation is an important epigenetic mechanism that causes gene silencing and has been linked to colon cancer [16,17]. To date, the role of the *BRAF* ^p.V600E^ mutation in DNA hypermethylation as found in colon tumors remains unclear.

The aim of this study was to analyze our previous dataset of *BRAF* ^p.V600E^ and *BRAF* wild type tumors [18] at the level of single genes. The most significantly hypermethylated gene in *BRAF* ^p.V600E^ colon tumors was the homeobox gene *MEIS1*. We explored the relationship between *MEIS1* promoter methylation and the *BRAF* ^p.V600E^ mutation in additional cohorts. We show that *MEIS1* methylation occurred more frequently in *BRAF* ^p.V600E^ mutated colon tumors, and that it corresponded with decreased *MEIS1* gene expression.

## Materials and Methods

### Ethics statement

Specific need for ethics committee’s approval was not necessary for this study. All samples were handled according to the medical ethical guidelines described in the Code Proper Secondary Use of Human Tissue established by the Dutch Federation of Medical Sciences (www.federa.org, accessed October 27, 2010). According to these guidelines all human material used in this study has been anonymized since clinical data were not used. Because of this anonymization procedure individual patients’ permission is not needed.

### Inclusion of patients

The first set of snap-frozen colorectal tumors meant for genome-wide differential methylation screening, originated from 19 anonymized patients with sporadic right-sided colon cancer were included who underwent surgery between 2002-2005 at the Leiden University Medical Center (Leiden, The Netherlands) or at the Rijnland Hospital (Leiderdorp, The Netherlands), as described previously [18]. Only patients of whom both tumor and corresponding normal tissue was available were further included.

A second, independent set of colorectal tumors was included for replication of results found in the first data set and consisted of 228 sporadic CRC patients who were operated between 1990 and 2005 at the Leiden University Medical Center. The third independent set consisted of 14 stage III colorectal tumors, of which aneuploid tumor cells and normal stromal cells were flow-sorted [19].

### Cell culture

Colon cancer cell lines (HCT15 [20], HT29, Caco-2 and LS180 [21], LoVo [22], LS411N [23], RKO [24], SW48, SW480, SW837 and SW1463 [25], Colo320DM [26] and T84 [27]) were obtained from the cell line collection of the department of Pathology at Leiden University Medical Center (Leiden, The Netherlands). Mutation analysis and short tandem repeat marker profiling confirmed their identity. Cell lines were cultured at 37°C in T75 flasks (Costar, Cambridge, UK) with RPMI-1640 medium supplemented with 10% Fetal Bovine serum, 2 mM Glutamax-I, 50 U Penicillin/mL medium and 50 µg Streptomycin/mL medium (GIBCO, Invitrogen LTD, Paisley, UK). 

### Sample preparation

For the first set of 19 colorectal tumors, fresh-frozen tumor tissue was first macrodissected based on evaluation of hematoxylin and eosin (HE) stained slides to remove non-tumor tissue [18]. In the second set of 228 colorectal tumors, formalin-fixed paraffin embedded (FFPE) colon tumor tissue was collected as 0.6 mm-diameter punches with a tissue microarrayer (Beecher Instruments, Inc., Sun Prairie, WI) based on evaluation of HE-stained slides [28]. The third set of 14 colorectal tumors was obtained from a previous study [19]. For each case, three different fractions were available: (i) the whole tumor sample; this fraction contained more than 50 to 70% of tumor cells obtained through HE-guided macrodissection; (ii) the DNA aneuploid, keratin-positive epithelial subpopulation; (iii) the vimentin-positive normal, diploid subpopulation. To obtain flow-sorted fractions, cell suspensions from each tumor were simultaneously stained for the epithelial cell marker keratin, the stromal marker vimentin, and for DNA content (propidium iodide). The samples were subsequently flow-sorted, as previously described [29]. The vimentin-positive fraction contained the normal stromal cells and lymphocytes that were present in the tumor tissue. The aneuploid keratin-positive fraction contained the epithelial tumor cells and was devoid of lymphocytes. For one tumor, two different keratin-positive tumor subpopulations were identified (TS510t); these were studied separately.

### DNA isolation and *BRAF* mutation analysis

For both the cell lines and the first set of 19 patients, DNA was isolated based on phenol/chloroform extraction, followed by ethanol precipitation. DNA from the 19 colorectal tumors were hybridized on Agilent 244k human CpG island microarrays (Agilent Technologies, Santa Clara, CA, U.S.A.), as described previously [18]. For the replication set of 228 colorectal tumors and fractionation set of 14 colorectal tumors, DNA was isolated using the NucleoSpin^®^ Tissue kit (Machery-Nagel, Germany). The *BRAF* ^p.V600E^ mutation was detected using exon 15 based mutant-allele-specific PCR [30].

### RNA extraction and RT-qPCR

Total RNA was isolated from human and cell line samples with TRIzol (Invitrogen, Paisley, U.K.) according to the manufacturer’s instructions. Subsequently, total RNA was purified with RNeasy Midi columns (QIAGEN, Venlo, The Netherlands), including a DNAse incubation step. RNA integrity and quality were evaluated by gel electrophoresis and spectrophotometric analysis on a Nanodrop^®^ (Thermo Scientific). For cDNA synthesis a mix of 1-2 µg of RNA, 1-2 U RNAsin/µL (RNasin^®^ Ribonuclease Inhibitor, Promega, Leiden, The Netherlands), 2.5 ng oligodT/µL, 0.08 µg Random primers/µL, 1 mM dNTPs and 0.25 U AMV RT transcriptase/µL was incubated for 1 hour at 42°C in a final volume of 20 µL. Subsequently, 2 µL of 25x diluted cDNA originating from cell lines or 125x diluted cDNA originating from human samples was assayed with 1x SensiMixPlus Sybr mix (GCBiotech, Augsburg, Germany) and 0.1 µM Forward and Reverse primer in a final volume of 8 µL. 

Primer sequences for both RT-qPCR and methylation specific PCR (MSP), as well as the corresponding assays are shown in the Table S1. *MEIS1* primers were designed as intron spanning primers across exon 5 and 6, to recognize the full length gene. The primer set for *MEIS1*_*D27*_ only recognizes truncated *MEIS1*, i.e. when exon 7 and 9 are fused after skipping of exon 8 [31]. *MEIS1* and *MEIS1*_*D27*_ gene expression were corrected for the geometric mean of two housekeeping genes, *CPSF6* and *HNRNPM* [32]. The Ct-values for human samples varied between 28 and 38, whereas Ct-values for human cell lines varied between 25 and 37.

### Methylation-specific PCR

Methylation-specific PCR (MSP) was performed for *MEIS1* and *MLH1* promoter regions. Following phenol/chloroform based DNA extraction isolation from frozen tissue of both tumor and paired normal tissues, 200 ng of DNA was bisulfite converted using the EZ DNA Methylation-Gold™ Kit (Zymo Research, Irvine, CA, U.S.A.), and eluted in 15 µL of M-Elution Buffer. Subsequently, 1 µL out of 15 µL eluate was amplified with 0.5 µM of each primer set that makes a distinction between unmethylated (Um) and methylated (M) *MEIS1* and *MLH1*, in combination with 0.1 U/µL AmpliTaq Gold^®^ DNA Polymerase (Applied Biosystems) and 0.2 mM dNTP mix. Details regarding the primer sets and corresponding assays are shown in Table S1. For high-throughput analyses of *MEIS1* promoter methylation status in the consecutive set of 228 patients, the above mentioned *MEIS1*-MSP protocol was slightly modified by adding the DNA-binding dye SYTO9 (1:500), enabling a real-time PCR based analysis of *MEIS1* methylation status.

### Statistical analyses

Agilent microarray data of paired tumor and normal tissue were processed in R2.10.0 (Bioconductor), as previously described [18]. Briefly, within-array normalization was performed with LOESS, followed by between-array normalization using Limma v3.2 [33], resulting in log_2_ ratios of tumor versus normal. Since the amplicon generation was based on MseI digestion, array probes were mapped to MseI fragments. For fragments with more than one mapped probe, the probe with the median log_2_ ratio was chosen as representative for the fragment. A linear model in Limma [34] was used to select differential methylation between *BRAF* wild type and mutant groups with a false discovery rate (FDR) ≤ 0.001 [35]. DNA methylation array data were deposited in the Gene Expression Omnibus (GEO) under accession number GSE39334.

The association between *BRAF* ^p.V600E^ and *MEIS1* promoter methylation was tested in a consecutive set of 228 patients by univariate logistic regression. The *BRAF* ^p.V600E^ associated variables right-sided tumor location and MSI-H were also tested for association with *MEIS1* promoter methylation. Additionally, *MEIS1* promoter methylation as a function of the interaction of these three variables was tested with multivariate logistic regression, using R2.16. The odds ratios (OR) and 95% confidence interval (CI) were reported.

## Results

### Patients

For this study, three independent sets of colorectal tumors were employed. For single gene analyses of DNA methylation profiling and validation, 19 paired tumor-normal samples were selected on presence in the ascending colon, including 8 *BRAF* ^p.V600E^ (6 of which were MSI-H and 2 MSS) and 11 *BRAF* wild types (9 of which were MSS and 2 MSI-H). Additional tumor characteristics, including histology, mismatch repair status, CpG island methylator phenotype (CIMP), *KRAS* and *p53* mutation status and *MLH1* methylation status are shown in Table S2 and were described previously [18]. 

Secondly, the association between *MEIS1* promoter methylation and *BRAF* ^p.V600E^ as initially found with CpG island microarray analyses was studied in a consecutive series of 228 colorectal tumors consisting of 54% males and an average age of 66 ± 12 years (mean ± SD). Tumors were tested for *MEIS1* promoter methylation status (*n* = 228), MSI status (*n* = 213), *BRAF* mutation status (*n* = 163) and tumor location (*n* = 168). The overlap between these sub-divisions is shown in Figure S1. 

Thirdly, the *MEIS1* promoter methylation status was determined in flow-sorted epithelial and stromal fractions of 14 patient samples that were part of a previous study [19] and shown in Table S3.

### Differential methylation of *BRAF* ^p.V600E^ compared to *BRAF* wild types

**Figure 1:**
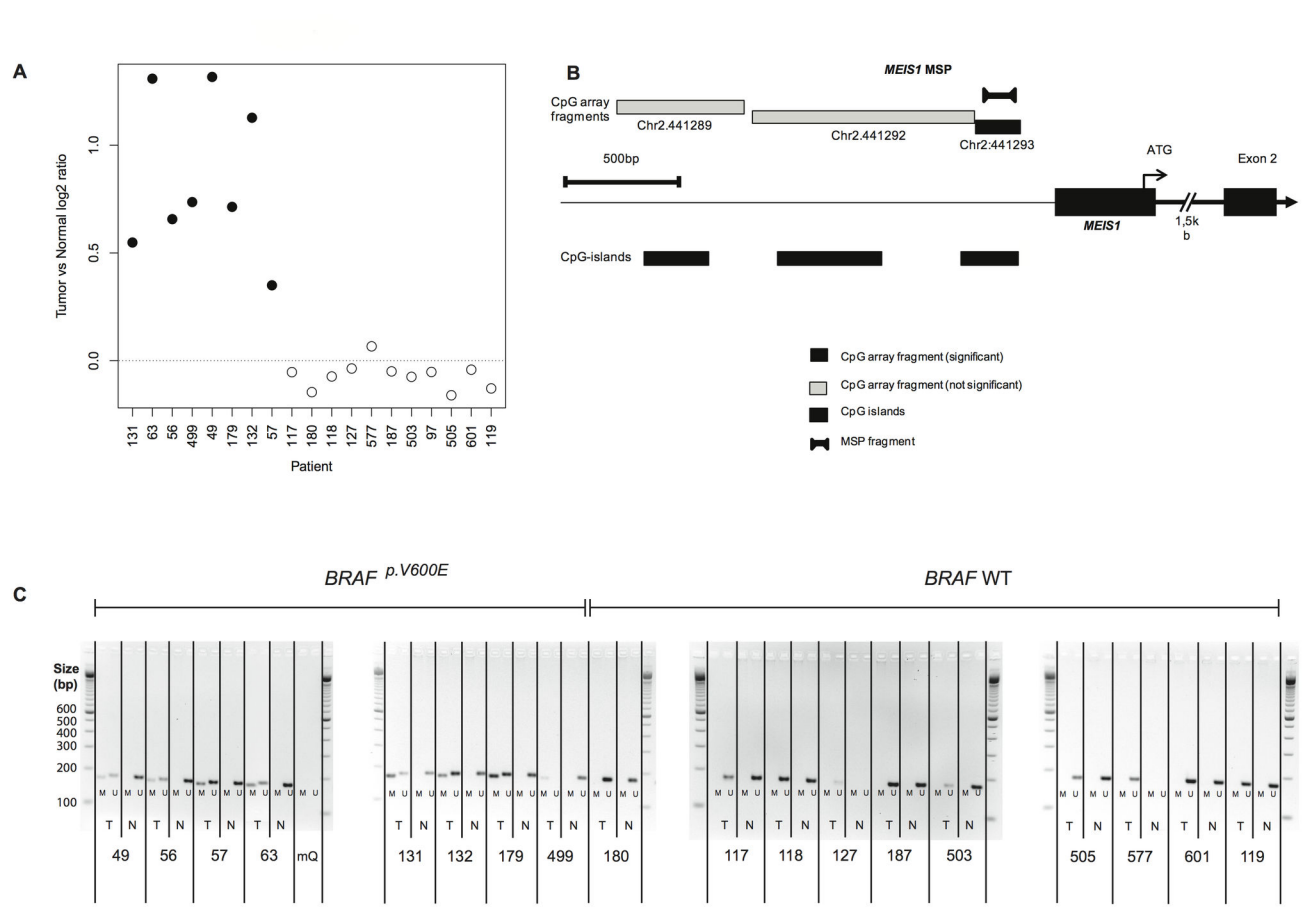
*MEIS1* is methylated in *BRAF* ^p.V600E^ relative to *BRAF* wild type tumors. (**A**) The *MEIS1* promoter is hypermethylated in colorectal tumors with a *BRAF* ^p.V600E^ mutation (black dots) when compared to wild type *BRAF* (white dots). The Y-axis represents the tumor vs. normal log_2_ ratio for the median probe per CpG fragment. The horizontal dotted line at log_2_ ratio 0 indicates an equal extent of *MEIS1* methylation in tumor and normal samples. (**B**) Overview of the analyzed *MEIS1* promoter, CpG islands within the promoter and the locus analyzed by MSP primers. Locations were based on the human genome browser (UCSC assembly March 2006, hg18). (**C**) *MEIS1*-MSP data showing hypermethylation in *BRAF* ^p.V600E^ colorectal tumors when compared to *BRAF* wild types. T: tumor; N: normal tissue; M: methylated *MEIS1* promoter (168 bp); Um: Unmethylated *MEIS1* promoter (176 bp).

Tumor versus paired normal methylation ratios were compared between *BRAF* ^p.V600E^ and *BRAF* wild types to identify *BRAF* ^p.V600E^ specific DNA methylation. In total, 210 fragments associated with 200 unique genes were differentially methylated (FDR ≤ 0.001). The top 10 of most significantly differentially methylated loci all showed high methylation ratios in *BRAF* ^p.V600E^ colorectal tumors (Table 1). The highest significance was found for *MEIS1* (log_2_ fold change: 0.89, FDR: 2.8*10^-9^), showing a ± 700x higher significance than the second differentially methylated gene. The top differentially methylated region mapped to the *MEIS1* promoter region, located 300 bp upstream from the transcription start site. *BRAF* ^p.V600E^ tumors unequivocally showed elevated *MEIS1* methylation levels when compared to *BRAF* wild types which showed an approximately equal extent of methylation in both tumor and paired normal colon tissue (Figure 1A). 

**Table 1:** Top 10 of most significantly differentially methylated loci in a *BRAF* ^p.V600E^ vs. *BRAF* wild type comparison.

**Gene Name**	**Fragment**	**Fragment start**	**Fragment end**	**Description**	**Log_2_ Fold change**	**FDR-adjusted *P*-Value**
*MEIS1*	Chr2.441293	66,515,620	66,515,851	Promoter	0.89	2.79 * 10^-9^
*POU6F2*	Chr7.269627	39,420,336	39,420,779	Inside	0.67	1.92 * 10^-6^
*NR4A3*	Chr9.573325	101,627,226	101,627,606	Promoter	0.74	2.68 * 10^-6^
*ISLR2*	Chr15.339570	72,209,856	72,210,394	Promoter	0.59	5.03 * 10^-6^
*GALR2*	Chr17.364837	71,582,754	71,583,229	Inside	0.70	5.61 * 10^-6^
*LYPD1*	Chr2.842595	133,144,875	133,145,523	Promoter	0.45	5.61 * 10^-6^
*COL4A2*	Chr13.724520	109,758,900	109,759,172	Inside	0.37	9.38 * 10^-6^
*SHC4*	Chr15.179740	47,042,094	47,042,663	Inside	0.63	9.38 * 10^-6^
*C1orf164*	Chr1.183112	44,854,835	44,855,693	Inside	0.34	9.42 * 10^-6^
*SYPL2*	Chr1.665313	109,810,333	109,810,621	Promoter	0.63	1.00 * 10^-5^

Fragment start and end position were retrieved from to the human genome browser (UCSC assembly March 2006, hg18). Fragments with the Description “unknown” were excluded from further analysis.

### Validation of *MEIS1* promoter methylation

MSP was employed to validate *MEIS1* promoter methylation, as found on the Agilent microarrays. An overview of the tested *MEIS1* region is given in Figure 1B. MSP performed on the same array-hybridized samples confirmed that *MEIS1* methylation was exclusively found in *BRAF* ^p.V600E^ tumors, whereas *BRAF* wild type tumors were unmethylated (Figure 1C). Furthermore, the normal tissue associated with both *BRAF* ^p.V600E^ and *BRAF* wild type tumors only showed unmethylated *MEIS1*. As a positive control for DNA quality and bisulfite conversion, we determined *MLH1* methylation in the same samples by MSP. The results were concordant with the mismatch repair status of these sporadic tumors, and with the DNA methylation array data for *MLH1* (Figure S2 A-C), suggesting high quality of bisulfite converted DNA. These data confirm that the *MEIS1* promoter is methylated in *BRAF* ^p.V600E^ colon tumors, but unmethylated in both normal tissue and *BRAF* wild type colon tumors.

### Validation of *MEIS1* promoter methylation in a consecutive cohort

To exclude the possibility that the correlation between *BRAF* ^p.V600E^ and *MEIS1* promoter methylation is a cohort specific effect, we analyzed an independent consecutive series of 228 colorectal tumors. The *MEIS1* promoter methylation status was determined by MSP, and analyzed in relation to *BRAF* mutation status, MSI status, and tumor location (Figure S1). In total, 18% of samples carried *BRAF* ^p.V600E^, 12% were MSI-H and 33% were located in the proximal colon. In *BRAF* ^p.V600E^ tumors 60% (18 out of 30) of the samples were methylated for *MEIS1*, while *BRAF* wild type tumors showed 13% (17 out of 133) *MEIS1* methylation. Univariate analysis, which considers individual variables contributing to *MEIS1* promoter methylation (*BRAF* mutation status, MSI status, and tumor location), showed that *BRAF* ^p.V600E^ had the highest association with *MEIS1* promoter methylation (OR = 13.0, CI = 5.2 - 33.0, *P* = 0.0001; Table 2). Lower associations were found between MSI-H and *MEIS1* promoter methylation (OR = 6.9, CI = 2.4 - 19.7, *P* = 0.0003), and between tumor location and *MEIS1* promoter methylation (OR = 2.4, CI = 1.1 - 5.4, *P* = 0.028). 

**Table 2:** Associations between *MEIS1* promoter methylation and *BRAF* mutation status, MSI and tumor location.

		**M**	**Um**	**Total**
***BRAF***	p.V600E	18	12	30
	WT	17	116	133
	Total	35	128	163
**MSI**	MSI-H	14	11	25
	MSS	31	157	188
	Total	45	168	213
**Location**	Proximal	17	39	56
	Distal	17	95	112
	Total	34	134	168

Number of patients involved in the calculation of the Odds Ratio for association analysis between *MEIS1* promoter methylation and *BRAF*, MSI and tumor location. WT: wild type; M: Methylated; Um: Unmethylated; MSI-H: Microsatellite Instable High; MSS: Microsatellite Stable. Proximal: right-sided tumors; distal: left-sided tumors.

Since *BRAF* ^p.V600E^ correlates with MSI-H and right-sided tumor location within the colon [3,14], multivariate logistic regression was performed to unravel possible associations between these three variables. *BRAF* ^p.V600E^ was the only significant variable associated with *MEIS1* promoter methylation, after adjustment for tumor location and MSI status (Adjusted OR = 10.2, CI = 3.7 - 27.7, *P* < 0.000001). For both MSI status (Adjusted OR = 2.8, CI = 0.7 - 11.0, *P* = 0.7) and for tumor location (Adjusted OR = 1.0, CI = 0.4 - 3.0, *P* = 0.5), no association was found with *MEIS1* methylation after adjustment for the remaining two variables. 

We conclude that the *BRAF* ^p.V600E^ mutation has the highest association with *MEIS1* promoter methylation. The frequency of *MEIS1* methylation among *BRAF* mutant tumors in the consecutive cohort with 228 patients was lower than in the discovery cohort with 19 patients, possibly due to the small size and selection for proximal colon of the discovery samples.

### *MEIS1* promoter methylation is associated with decreased *MEIS1* gene expression

**Figure 2:**
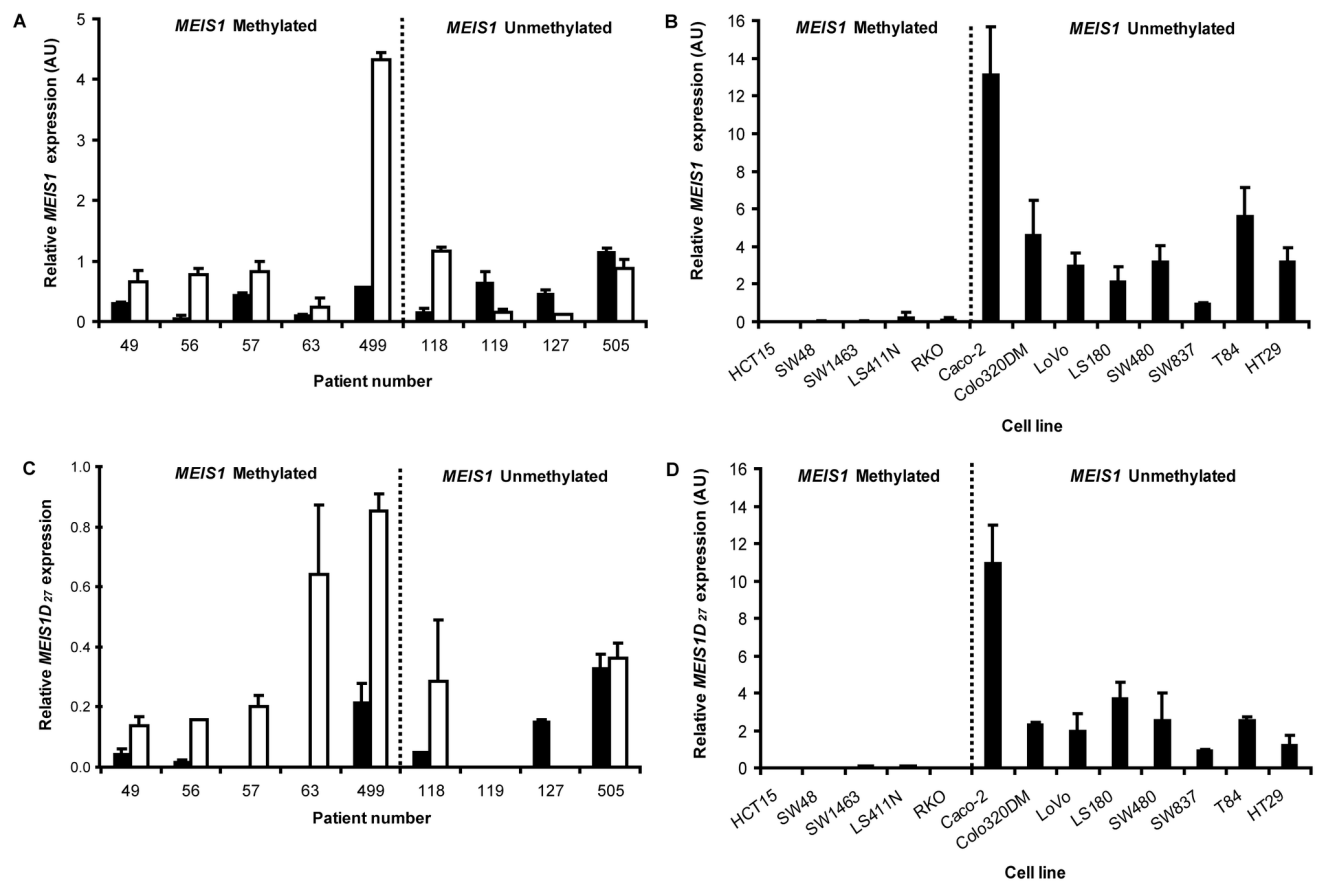
Gene expression of *MEIS1* and *MEIS1D*_*27*_ expression in human colorectal samples and CRC cell lines. *MEIS1* gene expression as measured by RT-qPCR corrected for the geometric mean of the housekeeping genes *CPSF6* and *HNRNPM*. (**A**) *BRAF* ^p.V600E^ tumors (black bars) that were all *MEIS1* methylated showed lower expression of the full length *MEIS1* gene, when compared to the paired normal tissues (white bars). (**B**) Colorectal cancer cell lines that were methylated for the *MEIS1* gene promoter were devoid of *MEIS1* gene expression. HT29, LS411N and RKO colon cancer cell lines carried the *BRAF* ^p.V600E^ mutation, whereas the remaining cell lines were wild type for *BRAF*. (**C**) Gene expression of the truncated *MEIS1D*_*27*_ transcript in *BRAF* ^p.V600E^ tumors (black bars) was low to absent when compared to paired normal tissue (white bars). Primer sets used, uniquely detect the truncated transcript. (**D**) *MEIS1* methylated colon cancer cells were devoid of *MEIS*_*D27*_ (i.e. exon 8 skipped *MEIS1*). Gene expression was expressed relative to SW837.

*MEIS1* gene expression was studied by RT-qPCR in samples from nine patients with available RNA out of the set of 19 patients that were analyzed by DNA methylation microarrays. *MEIS1* expression in both normal and colon tumor samples was variable, possibly reflecting tissue heterogeneity and inter-individual variation. *MEIS1* promoter methylation was accompanied by relatively lower levels of *MEIS1* gene expression in the five *BRAF* ^p.V600E^ colon tumors compared with their corresponding normal paired tissue (ratios between 0.07 and 0.59, Figure 2A). In comparison, only one out of four tumors without *MEIS1* methylation had lower *MEIS1* expression in the tumor relative to the paired normal tissue. To exclude the effect of tissue heterogeneity, we also determined *MEIS1* methylation and expression in a panel of colorectal cancer cell lines (Figure 2B). All *MEIS1* methylated cell lines were devoid of *MEIS1* gene expression, whereas unmethylated cell lines did show *MEIS1* gene expression. Interestingly, low levels of *MEIS1* expression were detected in cell line LS411N, which showed hemi-methylated *MEIS1*. 

In a recent study, the truncated transcription variant of *MEIS1*, *MEIS1D*_*27*_, showed decreased expression in the proximal colon, suggesting a tumor suppressor function [36]. In line with the decreased expression of full length *MEIS1*, *MEIS1D*_*27*_ expression was unequivocally decreased in five *BRAF* ^p.V600E^ patients with *MEIS1* promoter methylation relative to the paired normal tissue, and in one tumor with unmethylated *MEIS1* (Figure 2C). Also, the epithelial colorectal cancer cell lines with *MEIS1* methylation showed absence of *MEIS1D*_*27*_ expression (Figure 2D). The RT-qPCR results both in primary tissues and cell lines suggest that *MEIS1* promoter methylation leads to decreased *MEIS1* gene expression.

### *MEIS1* methylation in tumor and stromal cells

To determine the origin of *MEIS1* promoter methylation in heterogeneous tissue, we studied flow-sorted epithelial tumor cells and normal stromal fractions. After flow-sorting, keratin-positive epithelial tumor fractions and vimentin-positive stromal fractions of 14 colorectal tumors were successfully analyzed, of which eight were *BRAF* ^p.V600E^ and six were *BRAF* wild type (Table 3). Amongst the *BRAF* ^p.V600E^ tumors, three epithelial fractions showed *MEIS1* promoter methylation. Of these cases, TS510t showed tumor heterogeneity, where two aneuploid tumor fractions were isolated from the same tumor tissue. One of the tumor fractions showed *MEIS1* methylation, the other fraction was unmethylated for *MEIS1* and also the entire normal stromal fraction showed *MEIS1* promoter methylation. For tumor TS234t, *MEIS1* methylation in the tumor cells was assumed since methylation was detected in the complete tumor only and not in the flow-sorted stromal fraction. In total, four out of eight *BRAF* ^p.V600E^ tumor epithelial fractions (50%) showed *MEIS1* promoter methylation. Furthermore, three out of eight *BRAF* ^p.V600E^ tumor stromal fractions showed *MEIS1* promoter methylation (38%). Amongst the *BRAF* wild type tumors, one out of six showed *MEIS1* promoter methylation in both the epithelial and stromal fractions (17%).

**Table 3:** *MEIS1* promoter methylation status of colorectal tumors and associated fractions.

**Sample**	***BRAF***	**Tumor**	**Epithelial fraction**	**Stromal fraction**
TS234t	p.V600E	M	M*	Um
TS495t	p.V600E	M	M	Um
TS516t	p.V600E	M	M	M
TS510t	p.V600E	M	Um/M**	M
TS141t	p.V600E	-	Um	M
TS454t	p.V600E	Um	Um	Um
TS465t	p.V600E	Um	Um	Um
OX103t	p.V600E	Um	Um	Um
TS128t	WT	-	M	M
TS291t	WT	-	Um	Um
TS261t	WT	-	Um	Um
TS479t	WT	-	Um	Um
TS485t	WT	-	Um	Um
TS532t	WT	-	Um	Um

Overview of 14 stage III colorectal tumor samples that were flow-sorted and labeled as either epithelial (keratin-positive fraction) or stromal (vimentin-positive fraction) cells.

M: Methylated; Um: Unmethylated; WT: wild type.

* Not available, but most likely methylated taking into account that the complete tumor was methylated and stroma unmethylated.

** Two aneuploid epithelial fractions from the same tumor.

These data independently confirm that *MEIS1* promoter methylation has indeed occurred in the epithelial tumor cells of colon tumors carrying the *BRAF* ^p.V600E^ mutation. In addition, also the normal stromal cells from these tumors, which consisted of tumor infiltrating lymphocytes and fibroblast-like cells, showed *MEIS1* promoter methylation. 

## Discussion

Colorectal cancer shows molecular heterogeneity and accumulation of alterations at the level of both genetics and epigenetics, including DNA methylation. *BRAF* ^p.V600E^ mutated tumors are mainly located in the proximal colon, show MSI and relatively high DNA methylation levels [3,14]. Using a discovery cohort of 19 right-sided colon tumors and paired normal tissue, we found *MEIS1* as the most significantly hypermethylated gene promoter associated with *BRAF* ^p.V600E^ mutation. The association between *BRAF* ^p.V600E^ and *MEIS1* promoter methylation was validated in a larger, consecutive cohort and both significant when considering *BRAF* ^p.V600E^ as a single factor and after correction for MSI and right-sided tumor location. The frequency of *MEIS1* methylation in *BRAF* ^p.V600E^ mutated tumors was 60% for the consecutive cohort and 50% for the epithelial fractions of flow-sorted tumor samples. The lower frequency of *MEIS1* methylation in the validation cohorts compared with the discovery cohort (100%) is possibly due to the small size and selection for proximal colon of the discovery samples. In both validation cohorts, the frequency of *MEIS1* methylation in *BRAF* wild type tumors was low (13-17%). Therefore, we conclude that the association between *BRAF* ^p.V600E^ and *MEIS1* methylation is consistent. Strikingly, *MEIS1* is a highly expressed oncogene in leukemia [37], and its downregulation is a marker that indicates a good prognosis [38].

*BRAF* ^p.V600E^-associated *MEIS1* methylation was associated with decreased gene expression of the full length *MEIS1* transcript and a truncated isoform, *MEIS1D*_*27*_ in tumors and colon cancer cell lines. In line with our data, a previous study also showed decrease of *MEIS1* expression in colorectal adenomas [39]. The previously reported truncated *MEIS1* isoform that lacks exon 8, which is part of the DNA binding homeodomain, was shown to be expressed exclusively in the cytoplasm of epithelial cells in the right-sided colon [36]. The expression of this *MEIS1D*_*27*_ was decreased in colon tumors, when compared to paired normal tissue [36]. Since the *BRAF* mutation status of these colon tumors was not reported, it is not possible to evaluate whether loss of *MEIS1D*_*27*_ was associated with *BRAF* ^p.V600E^. 

Using flow-sorting, we detected *MEIS1* promoter methylation both in the epithelial tumor fractions as well as in the normal stromal fractions. The presence of *MEIS1* methylation in the epithelial fractions in tumors of which the stromal cells were not methylated, suggests a genuine role for *MEIS1* methylation in colorectal tumorigenesis. However, this finding is obscured by the presence of *MEIS1* methylation in the (normal) tumor stroma. We hypothesize that *MEIS1* methylation in the stroma may originate from infiltrating T-lymphocytes, similar to the methylation of *CDH1* in breast cancer [40]. These immune cells express vimentin [41] and were shown to acquire *MEIS1* methylation early during hematopoietic differentiation [42]. Additionally, intra-tumor lymphocyte infiltrate in colon tumors is associated with *BRAF* ^p.V600E^ [43] and MSI [44–47]. It should be noted that the CpG Island Methylator Phenotype (CIMP) is associated with *BRAF* ^p.V600E^ [15], which might imply that the CIMP status could be a confounder in the association between the *BRAF* mutation status and *MEIS1* methylation.

In conclusion, *MEIS1* methylation is associated with *BRAF* ^p.V600E^ in colon tumors and accompanied by a decrease of *MEIS1* gene expression. Further research is necessary to study the biological role of *MEIS1* in colon carcinogenesis, especially with a *BRAF* ^p.V600E^ mutation.

## Supporting Information

Figure S1 **The homeobox gene MEIS1 is methylated in BRAF p.V600E mutated colon tumors.**
(PDF)

Figure S2 ***MLH1*-MSP as a positive control for bisulfite-converted DNA.**
(PDF)

Table S1 **Primers and protocols used for PCR-based analyses.**
(DOCX)

Table S2 **Characteristics of the first set of 19 included patients.**
(DOCX)

Table S3 **Characteristics of patients selected for flow-sorting.**
(DOCX)
