# Supplementary material for: The Homeobox Gene MEIS1 Is Methylated in BRAF p.V600E Mutated Colon Tumors
Source: PLoS One. 2013 Nov 7;8(11):e79898. doi: 10.1371/journal.pone.0079898 (PMC3820613; doi:10.1371/journal.pone.0079898)
Supplement: Figure S1 — The homeobox gene MEIS1 is methylated in BRAF p.V600E mutated colon tumors. (PDF) [file pone.0079898.s001.pdf]

The homeobox gene MEIS1 is methylated in BRAF p.V600E mutated colon tumors.
Dihal et. al.

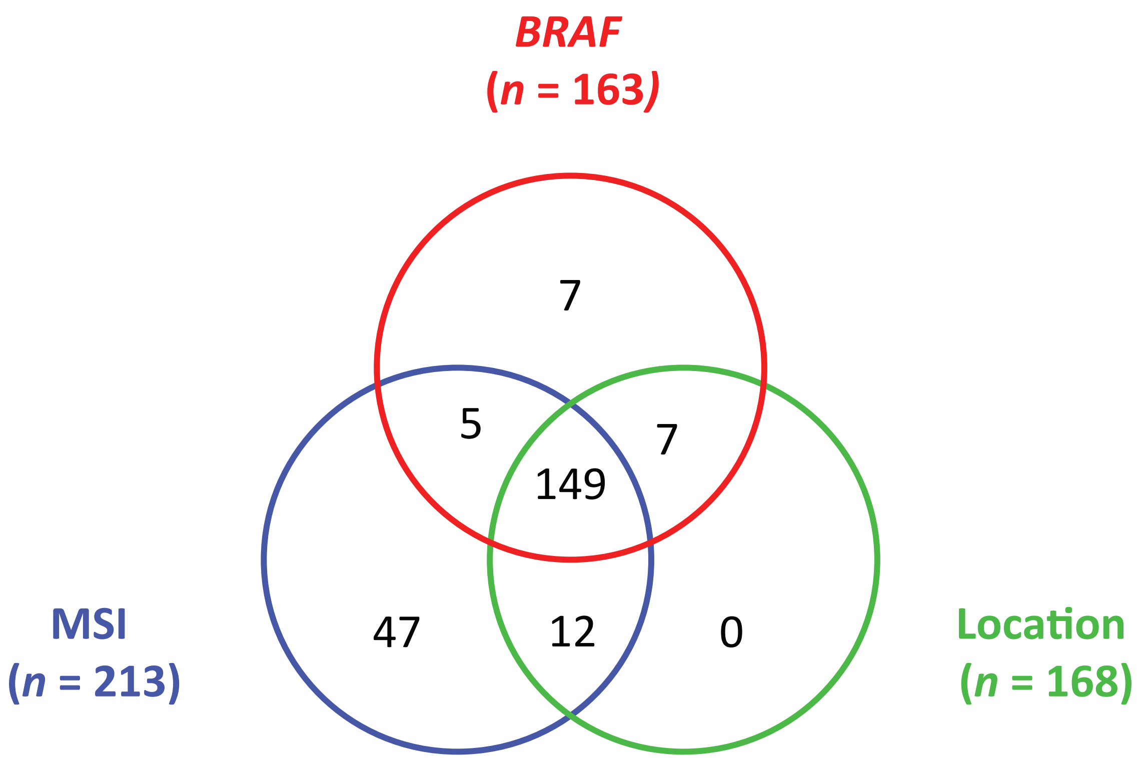

Figure S1

Venn diagram of patients with known MEIS1 methylation status (n = 228), meant for replication of MEIS1 promoter methylation data as found among the first set of 19 colorectal tumors.
Four out of fourteen patients in which the epithelial and stromal fractions were flow-sorted, overlapped with the above common denominator consisting of 149 patients.
MSI: Microsattelite Instable. Location: proximal (right-sided) or distal (left-sided) colorectal tumors.
