# Supplementary material for: The Homeobox Gene MEIS1 Is Methylated in BRAF p.V600E Mutated Colon Tumors
Source: PLoS One. 2013 Nov 7;8(11):e79898. doi: 10.1371/journal.pone.0079898 (PMC3820613; doi:10.1371/journal.pone.0079898)
Supplement: Figure S2 — MLH1-MSP as a positive control for bisulfite-converted DNA. (PDF) [file pone.0079898.s002.pdf]

Figure S2: *MLH1*-MSP as a positive control for bisulfite-converted DNA

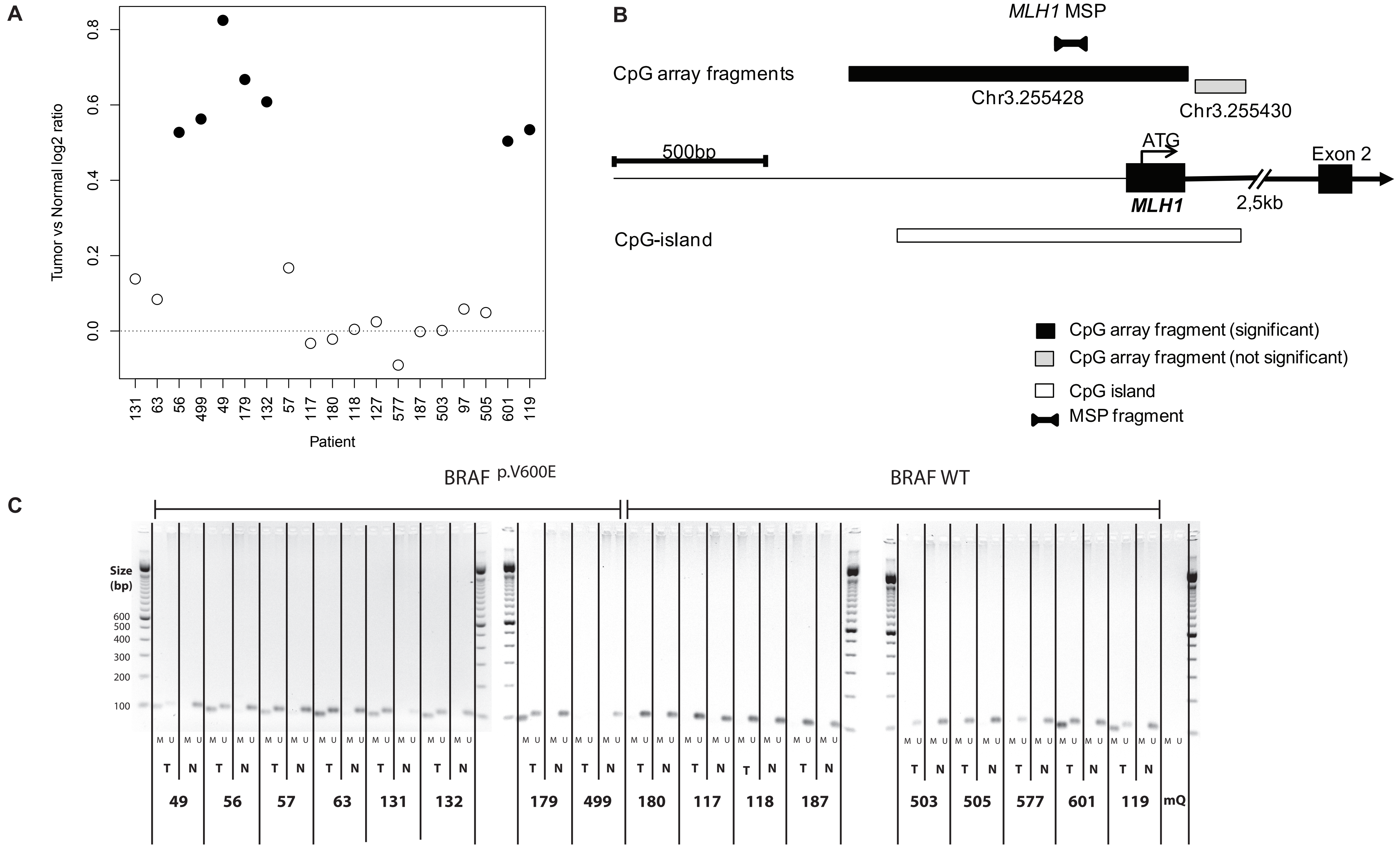

(A) *MLH1* promoter methylation (black dots) is spread amongst *BRAF p.*V600E mutation (first eight samples) when compared to wild type *BRAF* (last eleven samples) that were mainly *MLH1* unmethylated (white dots). The Y-axis represents the tumor vs. normal log2 ratio for the median probe per CpG fragment. The horizontal dotted line at log ratio 0 indicates an equal extent of *MEIS1* methylation in tumor and normal samples. (B) Overview of the analyzed *MLH1* promoter at 353 bp distance from the transcription start site, CpG islands within the promoter and the locus analyzed by MSP primers. Locations were based on the human genome browser (UCSC assembly March 2006, hg18). (C) *MLH1*-MSP validation data. Signals for both methylated and unmethylated *MLH1* are identical to data as found on the microarrays. T: tumor; N: normal tissue; M: methylated *MLH1* promoter (91 bp); Um: Unmethylated *MLH1* promoter (102 bp).
