# Supplementary material for: The Homeobox Gene MEIS1 Is Methylated in BRAF p.V600E Mutated Colon Tumors
Source: PLoS One. 2013 Nov 7;8(11):e79898. doi: 10.1371/journal.pone.0079898 (PMC3820613; doi:10.1371/journal.pone.0079898)
Supplement: Table S1 — Primers and protocols used for PCR-based analyses. (DOCX) [file pone.0079898.s003.docx]

**Supplementary Table S1. Primers and protocols used for PCR-based analyses (Dihal *et al*.)**

| **PCR** | **Primer** | **Primer sequences** | **Amplicon size (bp)** | **Protocol**  **Sec °C Cycles** | | |
| --- | --- | --- | --- | --- | --- | --- |
| **MSP** | *MEIS1*-FW (M) | 5’-CGTTTCGCGTATTTATTTTTGTC-3’ | 168 | 300 | 95 | 1 |
|  | *MEIS1*-RV (M) | 5’-GCTTACAATCCCCGTACGTA-3’ |  | 15 | 94 | 35 |
|  | *MEIS1*-FW (Um) | 5’-GAGTTTGTTTTGTGTATTTATTTTTGTTG-3’ | 176 | 30 | 58 | 35 |
|  | *MEIS1*-RV (Um) | 5’-CCCACTTACAATCCCCATACATA-3’ |  | 30 | 72 | 35 |
|  |  |  |  | 300 | 72 | 1 |
|  | *MLH1*-FW (M) | 5’-AACGAATTAATAGGAAGAGCGGATAGCG-3’ | 91 | 300 | 95 | 1 |
|  | *MLH1*-RV (M) | 5’-CGTCCCTCCCTAAAACGACTACTACCC-3’ |  | 15 | 94 | 33 |
|  | *MLH1*-FW (Um) | 5’-TAAAAATGAATTAATAGGAAGAGTGGATAGTG-3’ | 102 | 30 | 59 | 33 |
|  | *MLH1*-RV (Um) | 5’-AATCTCTTCATCCCTCCCTAAAACA-3’ |  | 30 | 72 | 33 |
|  |  |  |  | 420 | 72 | 1 |
| **RT-qPCR** | *MEIS1*-FW | 5’-TCAAGCCATACAAGTATTAAGGTTTC-3’ | 108 | 300 | 95 | 1 |
|  | *MEIS1*-RV | 5’-GGCATTTTCCCTTTCAAACA-3’ |  | 15 | 95 | 45 |
|  | *MEIS1D27*-FW* | 5’-AGCAGTGAGCAAGCACCCT-3’ | 134 | 30 | 60 | 45 |
|  | *MEIS1D27*-RV | 5’-agtgcagcccatgatagacc-3’ |  | 20 | 72 | 45 |
|  | *CPSF6*-FW** | 5’-AAGATTGCCTTCATGGAATTGAG-3’ | 89 | 420 | 72 | 1 |
|  | *CPSF6*-RV | 5’-TCGTGATCTACTATGGTCCCTCTCT-3’ |  |  |  |  |
|  | *HNRNPM*-FW** | 5’-GAGGCCATGCTCCTGGG-3’ | 85 |  |  |  |
|  | *HNRNPM*-RV | 5’-TTTAGCATCTTCCATGTGAAATCG-3’ |  |  |  |  |

MSP: Methylation-Specific Polymerase Chain Reaction. M: methylated; Um: Unmethylated

* The *MEIS1D27* primerset is designed to recognize the truncated *MEIS1* gene in which exon 8 is skipped and exon

7 and 9 are fused, as described by L. Xiong *et al*., Hum Mol Genet, 2009; p. 1065-74.

****  *CPSF6* and *HNRNPM* primersets were previously published by T. van Wezel *et al*., Breast Cancer Res, 2005, R998-1004.
