# Supplementary material for: The Homeobox Gene MEIS1 Is Methylated in BRAF p.V600E Mutated Colon Tumors
Source: PLoS One. 2013 Nov 7;8(11):e79898. doi: 10.1371/journal.pone.0079898 (PMC3820613; doi:10.1371/journal.pone.0079898)
Supplement: Table S2 — Characteristics of the first set of 19 included patients. (DOCX) [file pone.0079898.s004.docx]

**Supplementary Table S2. Characteristics of the first set of 19 included patients (Dihal *et al*.)**

| **Patient no.** | **Gender** | **Age (yr)** | **Histology** | **MMR status** | **CIMP status** | ***BRAF*** | ***KRAS*** | ***P53*** | ***MLH1*** | ***MINT1*** | ***MINT2*** | ***MINT12*** | ***MINT31*** | ***RIZ1*** | ***TIMP3*** | ***MINT27*** | ***Megalin*** |
| --- | --- | --- | --- | --- | --- | --- | --- | --- | --- | --- | --- | --- | --- | --- | --- | --- | --- |
| 117 | M | 69 | Ad | Proficient | - | WT | WT | mut | U | Um | Um | Um | Um | Um | Um | Um | Um |
| 180 | F | 86 | Ad | Proficient | - | WT | Mut | WT | U | Um/M | M | Um | Um | Um | Um | Um | Um |
| 118 | F | 69 | Ad | Proficient | - | WT | Mut | NA | U | Um | Um | Um | Um/M | Um | Um | Um | Um |
| 127 | F | 87 | Ad | Proficient | - | WT | Mut | NA | U | Um | Um | M | M | M | Um | Um | Um |
| 577 | M | 32 | Ad | Proficient | - | WT | Mut | NA | U | Um | Um | Um | Um | Um/M | Um | Um | Um |
| 187 | F | 61 | Ca | Proficient | - | WT | WT | mut | U | Um | Um | Um | Um | Um/M | Um | Um | Um |
| 503 | F | 63 | Ca | Proficient | - | WT | WT | mut | U | Um | Um | Um | Um | Um | Um | M | Um |
| 97 | F | 68 | Ca | Proficient | - | WT | Mut | mut | U | Um | Um | Um | Um | Um | Um | Um | Um |
| 505 | F | 83 | Ca | Proficient | + | WT | mut | mut | U | M | Um | M | M | M | Um | Um/M | Um |
| 601 | F | 83 | Ad | Deficient | + | WT | WT | NA | M | Um | Um | Um | Um | Um/M | Um | Um | Um |
| 119 | F | 76 | Ca | Deficient | + | WT | WT | WT | M | M | M | M | M | M | M | M | M |
| 131 | F | 71 | Ca | Proficient | + | Mut | WT | NA | U | M | M | M | M | Um/M | Um | M | Um |
| 63 | F | 57 | Ca | Proficient | + | Mut | WT | NA | U | M | M | M | M | M | M | Um/M | Um/M |
| 56 | M | 75 | Ca | Deficient | + | Mut | WT | WT | M | M | M | M | M | M | M | M | M |
| 499 | F | 69 | Ca | Deficient | + | Mut | WT | WT | M | M | M | M | M | M | M | M | M |
| 49 | F | 80 | Ca | Deficient | + | Mut | WT | WT | M | M | M | M | M | M | M | M | M |
| 179 | M | 90 | Ca | Deficient | + | Mut | WT | WT | M | M | M | M | M | M | M | M | M |
| 132 | F | 64 | Ca | Deficient | + | Mut | WT | NA | M | M | M | M | M | M | M | M | M |
| 57 | F | 75 | Ca | Deficient | + | Mut | WT | WT | U/M | Um | M | M | M | Um/M | Um/M | M | M |

Above data were published before by and modified from E.H. van Roon *et al.*, Clinical Epigenetics, 2013.

Ad: Adenoma; Ca: Carcinoma; CIMP: CpG Island Methylator Phenotype (- = CIMP negative and + = CIMP positive), WT: Wild type; NA: not available. M = Methylated; Um = Unmethylated
