# Supplementary material for: The Homeobox Gene MEIS1 Is Methylated in BRAF p.V600E Mutated Colon Tumors
Source: PLoS One. 2013 Nov 7;8(11):e79898. doi: 10.1371/journal.pone.0079898 (PMC3820613; doi:10.1371/journal.pone.0079898)
Supplement: Table S3 — Characteristics of patients selected for flow-sorting. (DOCX) [file pone.0079898.s005.docx]

**Supplementary Table S3. Characteristics of patients selected for flow-sorting**

**(Dihal *et al*.)**

| **Sample** | **MSI status** | ***K-Ras* status** | **Gender** | **Tumor location** | **Age (yrs)** |
| --- | --- | --- | --- | --- | --- |
| TS516 | MSS | WT | F | Proximal | 71 |
| TS234 | MSS | WT | M | Proximal | 66 |
| TS495 | MSS | WT | F | Proximal | 69 |
| TS510 | MSS | WT | M | Proximal | 72 |
| TS141 | MSS | WT | M | Proximal | 53 |
| TS454 | MSS | WT | F | Distal | 75 |
| TS465 | MSS | WT | F | Proximal | 73 |
| OX103 | MSS | WT | M | Proximal | 57 |
| TS128 | MSS | WT | M | Proximal | 61 |
| TS291 | MSS | WT | F | Proximal | 75 |
| TS261 | MSS | WT | M | Distal | 52 |
| TS479 | MSS | WT | M | Distal | 77 |
| TS485 | MSS | WT | F | Proximal | 71 |
| TS532 |  |  |  |  |  |

MSS: Microsatellite Stable

WT: Wild type

F: Female; M: male

Proximal: right-sided tumors

Distal: left-sided tumors
